# HPTLC Analysis and Chemical Composition of Selected *Melaleuca* Essential Oils

**DOI:** 10.3390/molecules28093925

**Published:** 2023-05-06

**Authors:** Aimé Vázquez, Nurhayat Tabanca, Paul E. Kendra

**Affiliations:** United States Department of Agriculture-Agricultural Research Service (USDA-ARS), Subtropical Horticulture Research Station (SHRS), Miami, FL 33158, USA; aime.vazquez@usda.gov (A.V.); paul.kendra@usda.gov (P.E.K.)

**Keywords:** tea tree oil, cajeput oil, nerolina oil, niaouli oil, rosalina oil, terpinene-4-ol, GC-MS, TLC

## Abstract

Tea tree oil (TTO) is a volatile essential oil obtained by distillation, mainly from the Australian native plant *Melaleuca alternifolia* (Maiden & Betche) Cheel (Myrtaceae). In this study, a comparative analysis of the chemical constituents of seven tea tree oils (*M. alternifolia*) and four other *Melaleuca* spp. oils (*M. cajuputi*, (M_Ca_), two chemotypes of *M. quinquenervia*, (M_Ne_ and M_Ni_), and *M. ericifolia* (M_Ro_)) was carried out using gas chromatography–mass spectrometry (GC-MS) and high-performance thin-layer chromatography (HPTLC). Among the seven TTOs, terpinen-4-ol (37.66–44.28%), γ-terpinene (16.42–20.75%), α-terpinene (3.47–12.62%), α-terpineol (3.11–4.66%), and terpinolene (2.75–4.19%) were the most abundant compounds. On the other hand, the most abundant compounds of the other *Melaleuca* oils varied, such as 1,8-cineole (64.63%) in M_Ca_ oil, (E)-nerolidol (48.40%) and linalool (33.30%) in M_Ne_ oil, 1,8-cineole (52.20%) in M_Ni_ oil, and linalool (38.19%) and 1,8-cineole (27.57%) in M_Ro_ oil. HPTLC fingerprinting of *Melaleuca* oils enabled the discrimination of TTO oils from other *Melaleuca* spp. oils. Variation was observed in the profile of the R_f_ values among EOs. The present study shows that HPTLC is one of the best ways to identify and evaluate the quality control in authenticating TTOs, other *Melaleuca* EOs, or EOs from other species within the Myrtaceae.

## 1. Introduction

Plant essential oils (EOs), originally used in the perfume and aromatherapy market, have gained widespread acceptance in other industries. Tea tree oil (TTO), for example, an essential oil isolated from *Melaleuca alternifolia* (Maiden & Betche) Cheel (Myrtaceae) [1], has long been recognized as a safe and effective topical antiseptic in Australia [2,3,4,5]. During World War II, it was considered a necessary commodity for first aid kits and was also used as an insect repellent [6]. Current commercial products that contain TTO include assorted ointments, lotions, shampoos, soaps, toothpastes, and mouthwashes [2,5,7]. Additionally, many EOs have since been evaluated for their antimicrobial [8,9], insect attractant [10,11], repellent [12], or insecticidal properties [13,14,15,16,17]. Isman [18] investigated the potential effect of several EOs as attractants, repellents, and toxicants against insects and other organisms.

Since EOs consist of concentrated plant terpenoids, they have provided an ideal substrate for the development of host-based (kairomone) lures for invasive pests in Florida, including the redbay ambrosia beetle *Xyleborus glabratus* Eichhoff, the tea shot hole borer *Euwallacea perbrevis* Schedl [19], and the Mediterranean fruit fly (medfly), *Ceratitis capitata* (Wiedemann) (Diptera: Tephritidae), which has been a destructive pest in Europe, the Middle East, Australia, Central and South America, and Hawaii, USA [20,21,22]. Behavioral studies conducted at the USDA-ARS in Miami, Florida [23,24], have indicated that tea tree oil (TTO) has a strong short-range attractive effect on sterile male medflies in laboratory bioassays [17,25,26,27,28,29,30,31]. Therefore, it has good potential as an economical new attractant for male medflies, and the identification of the key components of TTO is important for developing lures for pest management.

In recent experiments [27], we used preparative thin-layer chromatography (prep TLC) to separate TTO into five fractions. Bioassays conducted after these separations revealed that two TTO fractions are responsible for the observed attraction in male medflies. Furthermore, TLC-based bioassays played an important role in the isolation of insect kairomones from complex mixtures such as EOs. Although it is not a new technique, TLC continues to be a valuable tool as a preparative technique for a variety of studies in both chemical and biological fields [25,32,33,34,35]. While gas chromatography–mass spectrometry (GC-MS) is exceptional in the identification of unknown chemicals, it works best for trace analysis [36]. In addition, the sample is destroyed in the process of obtaining its fingerprint fragments for proper identification. In cases where a much larger sample is needed or it is required to remain intact for further analysis, TLC proves to be a more suitable method. With the development of high-performance thin-layer chromatography (HPTLC), this separation technique has evolved from a mostly qualitative procedure into a quick and cost-effective quantification method used by the European Pharmacopoeia for the quality control of some EOs [37]. The automated sample application, and the capability of using a universal HPTLC standard mix [38], ensures accurate sample and standard amounts, and provides reproducible quantitative results.

Various *Melaleuca* species, and even other Myrtaceae, are often confused under the common name ‘tea tree’, (e.g., ‘‘swamp tea tree’’, *M. cajuputi*; ‘‘paperbark tea tree’’, “broad-leaved tea tree”, or “broad-leaved paper bark”, *M. quinquenervia*; “black tea tree” or “river tea tree”, *M. bracteate*; “lemon scented tea tree”, *Leptospermum petersonii*, etc.). Moreover, kanuka and manuka EOs derived from Kunzea ericoides and Leptospermum scoparium, respectively, are referred to as New Zealand TTOs [2,3,5,6,39,40]. 

Considering the diversity of compounds present in *Melaleuca* EOs, and their current and potential applications, including prospective IPM strategies, it is important to study the chemical composition and degree of variability in commercially available *Melaleuca* EOs. In this study, HPTLC methods were developed for the evaluation of seven TTOs and four different *Melaleuca* spp. oils selected mainly from open markets in the United States. Separation patterns of various brands of TTO, as well as other *Melaleuca* oils, were compared to ensure the presence and consistent amount of chemicals attractive for male medflies.

## 2. Results and Discussion

### 2.1. Identification of Components in Melaleuca EOs

The identification of the components from the seven *M. alternifolia* EOs (TT_AA_, TT_AS_, TT_EG_, TT_FC_, TT_NG_, TT_PT_, and TT_SAT_) and four other *Melaleuca* EOs (M_Ca_, M_Ne_, M_Ni_, and M_Ro_) (Table 1) was achieved on the GC-MS using a non-polar DB-5 column. One hundred thirty-eight compounds were identified in total among the eleven *Melaleuca* EOs, accounting for 99.49-99.97% of the total composition (Table 2). 

The TTOs were characterized by a high amount of terpinen-4-ol (37.66–44.28%), followed by γ-terpinene (16.42–20.75%), α-terpinene (3.47–12.62%), α-terpineol (3.11–4.66%), terpinolene (2.75–4.19%), *p*-cymene (1.66–11.47%), α-pinene (1.29-2.66%), aromadendrene (1.20–1.90%), 1,8-cineole (1.17–4.79%), ledene (0.47–1.69%), and limonene (0.30–1.63%).

The GC-MS data indicated that the components of the other *Melaleuca* EOs varied notably from each other. The M_CA_ oil contained 1,8-cineole (64.43%) as a principal component, followed by α-pinene (5.48), α-terpineol (5.44%), β-caryophyllene (3.82%), γ-terpinene (3.24%), α-humulene (1.81%), β-selinene (1.28%), α-selinene (1.12%), β-pinene (1.11%), and terpinolene (1.04%). The most abundant constituents identified in the M_Ne_ oil were largely (*E*)-nerolidol (48.40%) and linalool (33.30%), whereas 1,8-cineole (52.20%), α-pinene (9.75%), (*E*)-nerolidol (6.89%), viridiflorol (6.23%), α-terpineol (5.08%), limonene (2.83%), β-caryophyllene (2.66%), β-pinene (2.56%), and ledene (1.76%) were identified as the main constitutes in the M_Ni_ oil. Linalool (38.19%) and 1,8-cineole (27.57%) were the main constituents of the M_Ro_ oil.

The International Standard, ISO 4730, requires terpinen-4-ol chemotype to be present in commercial TTO production [58]. ISO standards allow terpinen-4-ol between 30 and 48%, along with γ-terpinene (10–28%), α-terpinene (5–13%), 1,8-cineole (<0.01–15%), α-terpineol (1.5–8%), *p*-cymene (0.5–8%), α-pinene (1–6%), and terpinolene (1.5–5%), and containing a mixture of minor terpenoids with sabinene (<0.01–3.5%), aromadendrene (<0.01–3.0%), δ-cadinene (<0.01–3.0%), ledene (viridiflorene, <0.01–3.0%), limonene (0.5–1.5%), globulol (<0.01–1.0), and viridiflorol (<0.01–1.0%) [ISO]. Our GC-MS analysis revealed that all the TTO samples fitted into the terpinen-4-ol chemotype (37.7–44.3%) (Table 2). The therapeutic use of TTO is attributed to the concentration of terpinen-4-ol and 1,8-cineole (eucalyptol), yet 1,8-cineole has been reported to cause skin and mucous membranes irritation [59,60,61]. Therefore, low concentrations of 1,8-cineole are preferred to maximize the therapeutic use of TTO, and it is critical to distinguish between TTOs and other commercially available *Melaleuca* oils. 

*M. cajuputi* oil has three chemotypes. Chemotype 1 contains a high concentration (50–70%) of 1,8-cineole, while chemotype 2 contains a lower concentration of 1,8-cineole (31%), and chemotype 3 contains no 1,8-cineole. *M. cajuputi* subsp. *cajuputi* is the main source of cajuput oil, which does contain 1,8-cineole [61]. Our *M. cajuputi* (M_Ca_) oil from Thailand was found to be dominated by a 1,8-cineole-rich chemotype (Table 2).

*M. quinquenervia* can be a source of 1,8-cineole-rich essential oil [61]. Four chemotypes were reported for *M. quinquenervia* [62]; the cineole chemotype (1) contains 1,8-cineole (55.0–65.0%), α-pinene (7.0–12.0%), limonene (6.0–12.0%), α-terpineol (4.0-10.0%), β-pinene (1.5–4.5%), viridiflorol (1.0–3.5%), β-caryophyllene (0.01–2.0%), and myrcene (0.01–2.0%), and is called niaouli oil; the linalool chemotype (2) contains (*E*)-nerolidol (61.1%), linalool (23.9%), 1,8-cineole (2.6%), α-pinene (1.9%), terpinene-4-ol (1.8%), viridiflorol (1.6%), and β-caryophyllene (1.1%), and is called nerolina oil; the nerolidol chemotype (3) contains (*E*)-nerolidol (75.7–92.5%), β-caryophyllene (0.5–8.7%), 1,8-cineole (0.01–6.6%), caryophyllene oxide (0.1–6.1%), α-pinene + α-thujene (0–4.5%), δ-cadinol (0–2.5%), viridiflorol (0.1–1.7%), and α-terpineol + viridiflorene (ledene) (0-1.5%), and is also called niaouli oil; and the viridiflorol chemotype (4) contains viridiflorol (40.0–45.0%), 1,8-cineole (30.0–35.0%), (*E*)-nerolidol (3.0–6.0%), and ledol (0.01–4.0%), and is called niaouli oil, too. Our *M*. *quinquenervia* oil (M_Ni_) from Madagascar represents chemotype 1, and the oil (M_Ne_) from Australia represents chemotype 2 (Table 2). 

*M. ericifolia* is from native Australian plants and is known as the lavender tea tree or rosalina oil. The major compounds of rosalina oil were identified as linalool (35.0–55.0%), 1,8-cineole (18.0–26.0%), and α-pinene (5.0–12.0%) [61,62]. Our rosalina oil (M_Ro_) from Australia contained a high abundance of linalool and 1,8-cineole, followed by α-pinene (Table 2). Consequently, TTOs can be distinguished from other *Melaleuca* oils by the high amount of terpinene-4-ol and low amounts of 1,8-cineole and linalool, and the absence of (*E*)-nerolidol. 

TTO and other *Melaleuca* EOs were subjected to PCA and HCA in order to identify which constituents (detected at ≥0.5%) are different among the TTOs and the four other *Melaleuca* species. Principle components are reflected by eigenvalues. Table 3 shows that the seven components with eigenvalues greater than one account for 96.35% of the total variance. According to the rules of PCA, the highest eigenvalues, F1 (14.92) and F2 (8.60), were selected and then subjected to the PCA analysis. The Bartlett′s sphericity test carried out on the correlation matrix shows a calculated x^2^ = 1519.98, greater than the critical value x^2^ = 52.19 with 37 degrees of freedom (*p* < 0.0001), thus proving that PCA can achieve a significant reduction in the dimensionality of the original data set.

**Figure 1:**
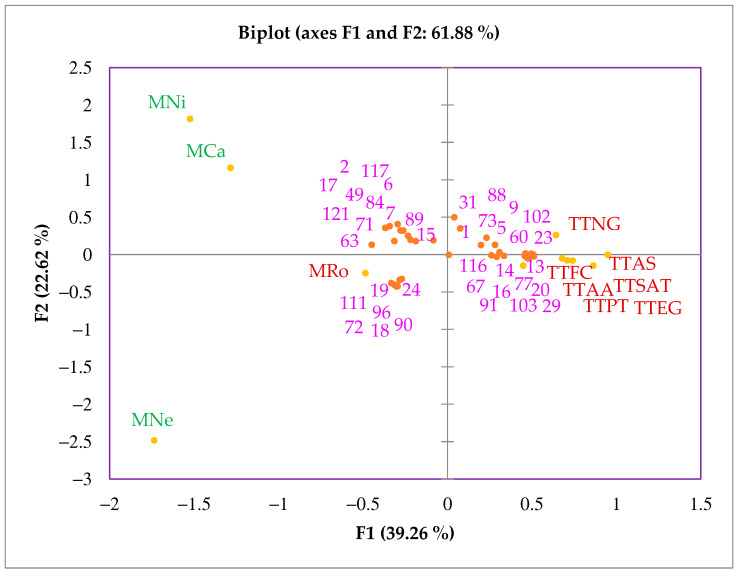
PCA biplot of TTO and other *Melaleuca* EOs based on their chemical composition. EO sample codes are listed according to the code reported in Table 1. The nomenclature of volatile compounds is listed in Table 2. Same color highlights similarity in chemical composition based on statistical analysis (See Section 3.5).

The PCA plot established according to the first two PCA axes is shown in Figure 1. Principal component 1 (F1, 39.26%) was strongly loaded for TTOs, with negative scores in M_Ca_, M_Ne_, M_Ni_, and M_Ro_. Principal component 2 (F2, 22.62%) demonstrated strong scores in M_Ca_, M_Ne_, M_Ni_, and low correlations with TTOs and M_Ro_, adding up to 61.88% of the variance in eleven *Melaleuca* EOs.

PCA provides a simple way to visualize similarities among different samples; a short range between the samples means a small or very little difference, and a long distance means a strong difference. Factor loadings and squared cosine (cos^2^) indicate the importance of components representing the individual components for a given principal component (Table 4). The cos^2^ similarity always tends to be 1, showing a high linear correlation relationship of the variables with the components. The highest cos^2^ values were in F1: γ-terpinene (0.902), terpinene-4-ol (0.900), terpinolene (0.886), δ-cadinene (0.844), *trans*-cadina-1(6),4-diene (0.828), bicyclogermacrene (0.798), α-gurjunene (0.798), zonarene (0.762), and α-terpinene (0.724). Similarly, the highest cos^2^ value for F2 was a-terpineol (0.858). When the relationship between the factor loadings and their percentage contributions to the matrix was analyzed, it could be concluded that α-thujene, α-pinene, β-pinene, 1,8-cineole, linalool, α-terpinyl acetate, α-terpineol, β-caryophyllene, α-humulene, (*E*)-β-farnesene, (*E*)-nerolidol, viridiflorol, and ledol had a negative relationship with the TTOs. Higher percentages of compounds then appear to be of interest for the selection of quality assessment of tea tree *M. alternifolia* EOs. 

**Figure 2:**
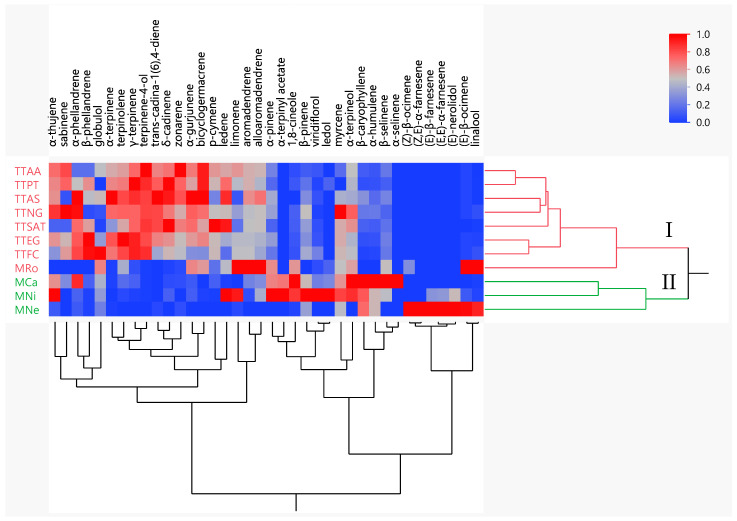
Two-way dendrogram of the hierarchical cluster analysis (HCA) performed on the chemical composition of the seven tea tree (*M. alternifolia*) EOs and four other *Melaleuca* EOs. Sample codes refer to Table 1. The color box indicated the abundance of each compound. Red represents high density and blue represents low density.

HCA classified Melaleuca EOs in two main groups (Figure 2): group I clustered samples with high contents of terpinene-4-ol, γ-terpinene, α-terpinene, and terpinolene. Although M_Ro_ did not belong to M. alternifolia (TTOs), it was grouped in cluster I due to its high levels of interfering compounds such as limonene, aromadendrene, and alloaromadendrene. The Euclidean distance between TTAA and TTPT was 1.74, and between TTAA and M_Ro_ it was 7.51. Group II clustered samples M_Ca_, M_Ni_, and M_Ne_, which presented high contents of 1,8-cineole, linalool, and (E)-nerolidol, with intermediate values of α-pinene, α-terpineol, and β-caryophyllene, and lower contents of terpinene-4-ol, γ-terpinene, terpineol, and α-terpinene, indicating that the TTOs contained significantly higher concentrations of terpinen-4-ol when compared to the other Melaleuca EOs.

### 2.2. HPTLC Analysis of Melaleuca EOs

**Figure 3:**
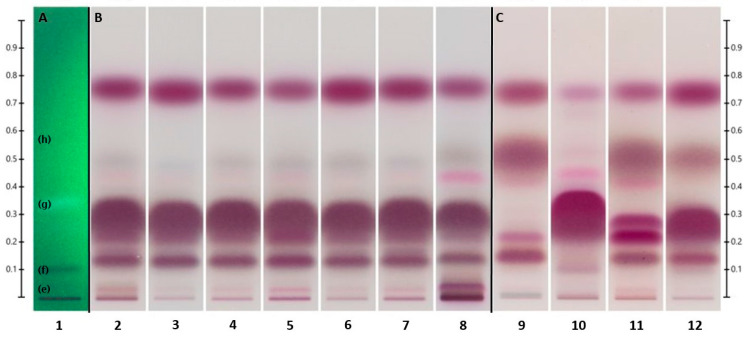
Separation of the UHM under UV_254_ (**A**) (see Table 5), tea tree oils (**B**), and other *Melaleuca* oils (**C**) (see Table 1) under visible light, developed with Hex/EtOAc 90:10 (*v*/*v*) on Silica gel 60 F_254_.

The less-polar components of the UHM, labeled (**e**) through (**h**) according to Do et al. [38], separated well under the selected HPTLC development conditions, as shown in Figure 3A. This indicated that the initial method involving Hex/EtOAc 90:10 (*v*/*v*) as the solvent system was appropriate for the separation of non-polar components of interest in our samples. However, the R_f_ of some *Melaleuca* oil components appear to exceed that of the highest UHM component. For future purposes, an additional component with higher R_f_ may need to be added to the UHM to better encompass the range of our samples.

In general, the HPTLC results complemented the findings obtained from the GC-MS analysis. The oils of *M. alternifolia* (TTOs) (Figure 3B), despite their wide variety of sources, exhibited a very similar separation pattern dominated by terpinen-4-ol as the primary component. When developed with Hex/EtOAc 90:10 (*v*/*v*), this main constituent of TTO appeared at an R_f_ value of 0.278 ± 0.067. A group of mono- and sesquiterpenes, including α-and β-pinene (R_f_ = 0.767 ± 0.031) and (+)-aromadendrene (R_f_ = 0.738 ± 0.062), was showcased as the second most prominent band, with a collective R_f_ of 0.747 ± 0.065. Included in this group were ledene and δ-cadinene, identified by GC-MS. This band was followed in intensity by α-terpineol (R_f_ = 0.147 ± 0.052) and 1,8-cineole (R_f_ = 0.504 ± 0.081), respectively.

In cases where oil components were merged, as observed by the overlapping or blending of colors, such components exhibited a shift in R_f_ values compared to those of the corresponding reference standards. This may be caused by interactions due to large amounts of components competing for the limited silica surface area. The R_f_ of the merged oil constituents, as well as the presence of additional constituents, was established by GC-MS. 

The other four *Melaleuca* EOs (Figure 3C) displayed a diverse pattern compared to each other, as well as in comparison to the TTOs. 1,8-cineole could be observed as the most characteristic component in M_Ca_ (track 9) and M_Ni_ (track 11), merged with small amounts of α-terpinyl acetate (R_f_ = 0.539 ± 0.050) and limonene (R_f_ = 0.486 ± 0.047). In contrast with M_Ca_ and M_Ni_, cineole was less prominent in M_Ro_ (track 12) and almost insignificant in M_Ne_ (track 10). No terpinyl acetate was observed in either M_Ne_ or M_Ro_, yet limonene was still present in M_Ro_. 

The second most noticeable band of M_Ca_ was a group of mono- and sesquiterpenes merged at R_f_ = 0.743 ± 0.052. These were identified by GC-MS as α- and β-pinene, β-caryophyllene (R_f_ = 0.727 ± 0.059), α-humulene (R_f_ = 0.727 ± 0.062), and α-phellandrene (R_f_ = 0.759 ± 0.031). Also identified by GC-MS in small amounts were α- and β-selinene. Two other chemicals present at a lower intensity, yet highly identifiable on M_Ca_, were α-terpineol and linalool (R_f_ = 0.243 ± 0.056).

The two most prominent bands in M_Ne_, combined near 0.3 R_f_, were identified as nerolidol (R_f_ = 0.279 ± 0.056) and linalool, respectively. They were followed in intensity by the mono- and sesquiterpene band near 0.75 R_f_, comprised of β-caryophyllene, β-pinene, β-ocimene (R_f_ = 0.747 ± 0.047), and farnesene (R_f_ = 0.737 ± 0.060), as identified by GC-MS. Small, yet still characteristic of M_Ne_, was geraniol at R_f_ = 0.124 ± 0.067. A bright-pink band at R_f_ = 0.432 ± 0.045 was recognized as caryophyllene oxide by isolating the component on a TLC preparation plate (Section 3.4.2) and confirmed by GC-MS analysis against its reference standard and available GC-MS libraries. Caryophyllene oxide appeared to be a byproduct of β-caryophyllene since it was present in both TLC and TIC reference standard chromatograms.

The M_Ni_ oil was characterized by a large amount of 1,8-cineole. As with M_Ca_, this large band was mixed with a small amount of terpinyl acetate and limonene. The second-largest band was composed of mono- and sesquiterpenes, confirmed by GC-MS as α- and β-pinene, β-caryophyllene, and ledene. Other main components were nerolidol, α-terpineol, and viridiflorol (R_f_ = 0.213 ± 0.021).

**Figure 4:**
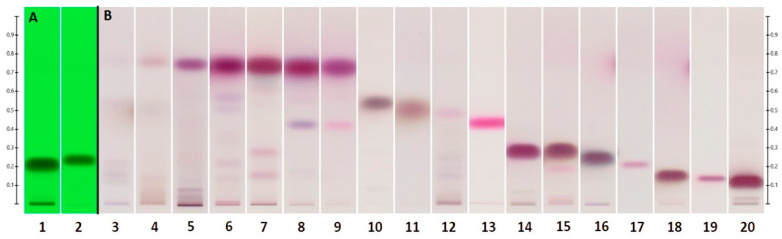
SSTs (**A**) are shown under UV_254_ light. Reference standards (**B**) (see Table 6) are shown under visible light. Developed with Hex/EtOAc 90:10 (*v*/*v*) on Silica gel 60 F_254_.

In M_Ro_, the signature component was linalool, with a small amount of terpinen-4-ol merging into it. Mono- and sesquiterpenes composed the second-strongest band near 0.76 R_f_, followed by cineole as the third characteristic band. Constituents of the second band were recognized as α-pinene, aromadendrene, alloaromadendrene, and ledene by GC-MS. Merged into a fourth band was α-terpineol, followed by globulol a bit below, at R_f_ = 0.137 ± 0.022. System suitability as well as R_f_ values for the major components were established by SSTs (Figure 4A) and reference standards (Figure 4B), analyzed under the same HPTLC conditions. 

**Figure 5:**
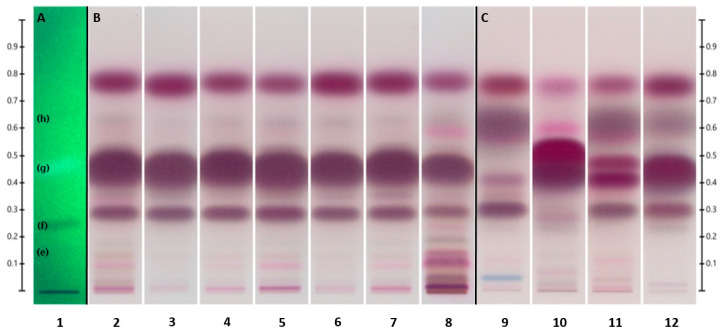
Separation of the UHM under UV_254_ (**A**) (see Table 5), tea tree oils (**B**), and other *Melaleuca* oils (**C**) (see Table 1) under visible light, developed with Hex/EtOAc 80:20 (*v*/*v*) on Silica gel 60 F_254_.

An enhanced separation of more polar constituents in the oils (lower R_f_ range) was obtained when developed with Hex/EtOAc at a ratio of 80:20 (*v*/*v*). Figure 5A emphasizes an increased distance among UHM constituents. Additionally, target oil components that merged at low R_f_ values under the previous solvent system were now appearing mid-range and better separated.

**Figure 6:**
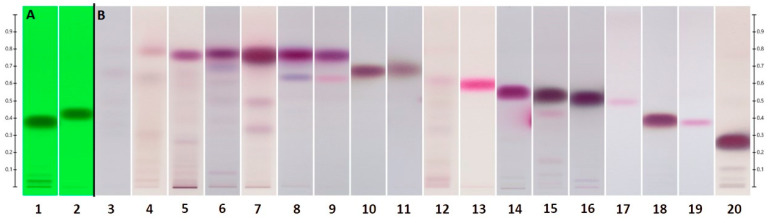
SSTs (**A**) are shown under UV_254_ light. Reference standards (**B**) (see Table 6) are shown under visible light. Developed with Hex/EtOAc 80:20 (*v*/*v*) on Silica gel 60 F_254_.

The most significant improvement with a more polar solvent system was an increased separation of terpinen-4-ol (R_f_ = 0.532 ± 0.049) and α-terpineol (R_f_ = 0.385 ± 0.054) in TTOs (Figure 5B). With the other *Melaleuca* oils (Figure 5C), where the differences among samples relied more on the polar region, a greater distinction could be made among nerolidol (R_f_ = 0.549 ± 0.052), linalool (R_f_ = 0.514 ± 0.055), viridiflorol (R_f_ = 0.494 ± 0.027), and terpinen-4-ol (R_f_ = 0.532 ± 0.049), as well as between α-terpineol (R_f_ = 0.385 ± 0.054), globulol (R_f_ = 0.376 ± 0.021), and geraniol (R_f_ = 0.263 ± 0.052). These differences in R_f_ were more evident at lower concentrations, as demonstrated by the SSTs (Figure 6A) and the individual reference standards (Figure 6B) analyzed under exact HPTLC developing conditions.

## 3. Materials and Methods

### 3.1. Sample Selection and Preparation

TTOs from *M. alternifolia* were selected based on their previously established biological activity as a potential attractant for the male Mediterranean fruit fly [23,24,26,27]. Essential oils from other *Melaleuca* species were also included for the purpose of comparison (Table 1). Each sample was diluted to 20% of its original purity using methylene chloride, ACS Reagent, CAS# 75-09-2 (J.T. Baker-Avantor, Center Valley, PA, USA). If necessary, concentration and application volume were adjusted for optimum HPTLC separation.

### 3.2. Standard Selection and Preparation

A universal HPTLC calibration mix (UHM) (Sigma-Aldrich, St. Louis, MO, USA) was used as a reference standard for HPTLC separations. It contains eight different compounds diluted in methanol at ready-to-use concentrations [38], four of which were observed under our HPTLC conditions using a UV_254_ light source (Table 5).

Stock solutions of isoeugenol, CAS# 97-54-1, and isoeugenyl acetate, CAS# 93-29-8 (Sigma-Aldrich, St. Louis, MO, USA), were prepared in methylene chloride at a concentration of 100 µL/mL and 100 µg/mL, respectively. A 20 µL/mL (21.6 mg/mL; δ = 1.08 g/mL) methylene chloride dilution of isoeugenol, as well as a 20 mg/mL dilution of isoeugenyl acetate, were prepared from their corresponding stock and used as system suitability standards (SST1 and SST2).

A series of reference standards (Table 6) were obtained from Sigma-Aldrich (St. Louis, MO, USA), and were prepared and analyzed under the same conditions as the EOs to confirm the R_f_ values of the oil components on HPTLC.

### 3.3. Gas Chromatography–Mass Spectrometry (GC-MS) Analysis

*Melaleuca* EO samples were analyzed on an Agilent 7890B GC coupled with a 5977B mass selective detector (GC-MS) (Agilent Technologies, Santa Clara, CA, USA). A DB-5 column (30 m × 0.25 mm inner diameter with 0.25 μm film thickness) was used with an electron ionization source set at 70 eV. The temperatures of the ion source and quadrupole were 230 °C and 150 °C, respectively. The mass spectrometry transmission line was 250 °C. Injector and detector temperatures were kept at 220 °C and 230 °C, respectively. The oven temperature program was set at 60 °C for 1.3 min and increased to 246 °C at 3 °C/min. A constant helium flow of 1.3 mL/min was applied [41]. The selected mass range was *m*/*z* 35 to 450 Da and scan rate was 2.8 scans/s. Mass Hunter B.07.06 software (Agilent Technologies) was used for data acquisition and processing. One μL of diluted samples was injected into the GC–MS on splitless mode. 

Linear retention indices (RIs) were calculated using the van Den Dool and Kratz [42] equation in relation to a homologous series of *n*-alkanes (C_9_–C_21_). Compound identification was achieved by comparison of their corresponding mass spectra and RIs to those reported in a mass spectral library developed at the USDA-ARS-SHRS laboratory with authentic compounds and with the commercial libraries MassFinder [43], Adams Library [41], Flavours and Fragrances of Natural and Synthetic Compounds 3 (FFNSC-3) [44], Wiley 12/NIST 2020 [45], and an in-house library “SHRS Essential Oil Constituents-DB-5 Column”. Retention indices were also verified with data reported in the specific literature [46,47,48,49,50,51,52,53,54] and internet sources [55,56,57]. Each oil was analyzed in triplicate. Relative percentages were directly obtained from peak total ion current (TIC) areas. All these standards were purchased from the following sources: α-pinene (CAS# 80-56-8), camphene (CAS# 79-92-5), benzaldehyde (CAS# 100-52-7), sabinene (CAS# 3387-41-5), β-pinene (CAS# 127-91-3), myrcene (CAS# 123-35-3), 6-methyl-5-hepten-2-ol (CAS# 1569-60-4), α-phellandrene (CAS# 99-792-5), δ-3-carene (CAS# 13466-78-9), 1,4-cineole (CAS# 470-67-7), α-terpinene (CAS# 99-86-5), *p*-cymene (CAS# 99-87-6), limonene (CAS# 5989-27-5), 1,8-cineole (CAS# 470-82-6), ocimene mixture (CAS# 13877-91-3), γ-terpinene (CAS# 99-85-4), linalool oxide (CAS# 60047-17-8), terpinolene (CAS# 586-62-9), linalool (CAS# 78-70-6), terpinen-4-ol (CAS# 20126-76-5), α-terpineol (CAS# 10482-56-1), citronellol (CAS# 106-22-9), geraniol (CAS# 106-24-1), thymol (CAS# 89-83-8), carvacrol (CAS# 499-75-2), α-terpinyl acetate (CAS# 80-26-2), eugenol (CAS# 97-53-0), geranyl acetate (CAS# 105-87-3), methyl eugenol (CAS# 93-15-2), β-caryophyllene (CAS# 87-44-5), aromadendrene (CAS# 489-39-4), α-humulene (CAS# 6753-98-6), (*E*)-β-farnesene (CAS# 18797-84-8), farnesene, mixture of isomers (product number W383902), alloaromadendrene (CAS# 25246-27-9), nerolidol (CAS# 7212-44-4), caryophyllene oxide (CAS# 1139-30-6), globulol (CAS# 489-41-8), viridiflorol (CAS# 552-02-3), α-bisabolol (Cas# 23089-26-2), farnesol mixture (CAS# 4602-84-0) from Sigma-Aldrich, St. Louis, MO, USA; β-phellandrene (CAS# 555-10-2) from Toronto Research Chemicals (Toronto, ON, Canada); α-copaene (CAS# 3856-25-5) and β-elemene (CAS# 515-13-9) from Fluka Chemical Co., Buchs, SG, Switzerland); and (+)-*ar*-curcumene (CAS# 4176-06-1) from BOC Sciences Shirley, NY, USA.

### 3.4. Thin-Layer Chromatography Analysis

#### 3.4.1. Automated High-Performance Thin-Layer Chromatography (HPTLC) Analysis

Chromatography was performed using a CAMAG HPTLC system equipped with VisionCATS 3.1 software (CAMAG, Muttenz, Switzerland). Initial conditions were set following the established HPTLC/TLC protocol for essential oils [37,63]. An HPTLC Silica gel 60 F_254_ glass-backed plate, 20 × 10 cm (Supelco Merck KGaA, Darmstadt, Germany, operating as Millipore-Sigma in St. Louis, MO, USA), was activated by heat using a TLC Plate Heater III (CAMAG, Muttenz, Switzerland) for 10 min. at 65 °C prior to analysis. Toluene/ethyl acetate 93:7 (*v*/*v*) was used as the mobile phase. However, previous bioassays (P.E.K. unpublished data) had indicated that sterile male medflies were repelled by toluene residue, prompting the search for an alternative mobile phase. Hexane was selected due to its similar polarity to toluene.

*Melaleuca* EO constituents appear in different amounts and cover a relatively wide polarity range when separated by TLC. Tabanca et al. [27] used hexane/acetone 90:10 (*v*/*v*) and obtained a good separation that produced two TTO fractions attractive to sterile male medflies, yet these fractions still contained a mixture of chemicals. Further separation was necessary to identify possible individual attractants. Various ratios of hexane/ethyl acetate were then attempted in preliminary experiments and it was decided that two separate solvent combinations provided improved resolution of the fractions of interest.

For the separation of monoterpenes and other non-polar oil constituents, a solution of 45 mL hexane (Hex), Certified ACS, CAS# 92112-69-1 (Fisher Chemical, Thermo Fisher Scientific, Waltham, MA, USA), and 5 mL ethyl acetate (EtOAc), HPLC grade, ≥99.7%, CAS#141-78-6 (Sigma-Aldrich, St. Louis, MO, USA), was prepared for a ratio of 90:10 (*v*/*v*). To favor the separation of more polar compounds, 40 mL Hex and 10 mL EtOAc were mixed for an 80:20 (*v*/*v*) ratio.

An aliquot of each oil sample was dispensed into a 1.5 mL screw-cap vial, covered with TFE/SIL septum cap (J.G. Finneran Associates, Inc., Vineland, NJ, USA), and placed into an Automatic TLC Sampler (ATS4) (CAMAG, Muttenz, Switzerland). An activated silica gel plate was placed in its corresponding holder. Samples were applied as thin bands (8 mm long, 8 mm from the bottom edge of the plate) using a 25 µL Hamilton syringe with spray application needle and nozzle. Syringe and needle were automatically rinsed 5 times with methanol, ACS grade, CAS# 67-56-1 (Supelco Merck KGaA, Darmstadt, Germany, as EMD Millipore Corporation, Burlington, MA, USA), between samples.

The HPTLC plate was developed in an Automatic Developing Chamber (ADC2) (CAMAG, Muttenz, Switzerland). The chamber containing a saturation pad was saturated for 20 min with 25 mL of the selected mobile phase. To remove as much moisture as possible, the system was also activated for 10 min with a saturated magnesium chloride aqueous solution prepared from magnesium chloride hexahydrate, (MgCl_2_.6H_2_O), CAS# 7791-18-6 (Sigma-Aldrich, St. Louis, MO, USA). Development was automatically started and stopped once the solvent front reached a preset height of 85 mm. After development, the plate was allowed to dry for 1 min at room temperature in the fume hood.

A vanillin/sulfuric acid derivatizing reagent was prepared following Wagner and Bladt [63], by adding 0.4 g of vanillin Reagent Plus, 99%, CAS# 121-33-5 (Sigma-Aldrich, St. Louis, MO, USA), to 100 mL of 190 proof ethanol, USP, CAS# 64-17-5 (Decon Labs, Inc., King of Prussia, PA, USA). The ethanolic solution was kept refrigerated until use. Concentrated sulfuric acid, Certified ACS Plus, CAS# 7664-93-9 (Fisher Chemical, Thermo Fisher Scientific, Waltham, MA, USA), was added shortly before use at a proportion of 20 µL acid per mL of vanillin solution. 

Automatic derivatization of the plate to generate color occurred inside a Derivatizer chamber (CAMAG, Muttenz, Switzerland) with 2 mL of vanillin/H_2_SO_4_ reagent. The derivatizing reagent was applied by spraying through a yellow nozzle at spray level 3. Colors were observed after heating the plate for 1.5 to 3.0 min at 100 °C on a CAMAG Plate Heater III, depending on color intensity. Images of the plate were taken at various stages of the process using a Visualizer 2 (CAMAG, Muttenz, Switzerland) with a 16 mm lens under RT White, UV_254_, and UV_366_ light. The retention factors (R_f_) values were calculated by VisionCATS software version 3.1. Profiles and comparisons were also generated using VisionCATS.

#### 3.4.2. Automated Preparative Thin-Layer Chromatography Analysis

Preparative TLC was used to isolate unknown bands for identification in cases where a component was not readily identified, and a standard could not be easily referenced for confirmation. An HPTLC Silica gel 60 F_254_ glass-backed plate, 20 × 10 cm (Supelco Merck KGaA, Darmstadt, Germany, operating as Millipore-Sigma in St. Louis, MO, US), was used to separate and collect a reasonable amount of the unknown chemical for further identification. The plate was initially activated by heating at 100 °C for 15 min on a CAMAG TLC Plate Heater III.

Sample application was conducted in a CAMAG ATS4 autosampler, where 2 µL oil was applied in 20 consecutive bands, 8 mm long each, making a solid horizontal line 8 mm from the bottom edge of the plate. Once the sample was applied, the plate was developed in a previously saturated CAMAG ADC2 chamber. A total of 35 mL mobile phase was used, 25 mL for saturation and 10 for development. Development stopped when solvent front reached 85 mm.

In the case of nerolina oil, a bright pink band at R_f_ = 0.451 ± 0.022 (40–45 mm from the bottom) was our target chemical. A 5 × 10 cm strip was cut out of the developed plate and was sprayed with vanillin reagent in a CAMAG Derivatizer. The bright pink band on the derivatized strip provided the measurements of the area to scrape to obtain our unknown from the remaining (non-derivatized) portion of the plate. Scraped silica containing our compound of interest was extracted with 1 mL methylene chloride and filtered through a 0.2 µm Whatman AUTOVIAL™ 5 syringeless filter (Global Life Sciences Solutions USA LLC–Cytiva, Marlborough, MA, USA) for GC-MS analysis.

### 3.5. Statistical Analysis

Principal component analysis (PCA) and hierarchical cluster analysis (HCA) were applied to TTOs and other *Melaleuca* EOs and their chemical constituents, using the XLSTAT 2021 (Addinsoft, New York, NY, USA) for PCA and JMP (JMP^®^ Pro 17.0.0, SAS Institute Inc. Cary, NC, USA) for HCA. Both PCA and HCA were performed on the means of those volatile constituents higher than 0.5%; the covariance data matrix was 38 × 11 (418 data). Pearson’s correlation model was used for PCA, Euclidean distance for measure, and Ward’s method for HCA analysis.

## 4. Conclusions

The results of this study demonstrate that HPTLC serves as a quick and effective analytical technique for the screening of selected *Melaleuca* oils. Its automated steps eliminate most human error and provide better reproducibility. A wide variety of samples may be analyzed by combining the most suitable mobile and stationary phases for the target analytes. This allows for the selection of less toxic solvents, such as hexane instead of toluene, while maintaining comparable retention factors to those in the Pharmacopeia. It also provides a fast detection tool for more polar additives or contaminants that may not be detected under GC-MS conditions. Samples can be applied as a long, narrow band, allowing multiple samples to be simultaneously analyzed and a cleaner separation of individual components.

An advantage over other analytical techniques is that multiple samples and standards may be analyzed at the same time and under true identical conditions using HPTLC. Moreover, the development process is nondestructive, which allows samples to be scraped and extracted from the plate for further studies. For this purpose, a template may be created from a prior plate derivatized with color reagent.

On the other hand, there are some disadvantages to this procedure. In the case of highly volatile constituents, there is a high probability of evaporation during the process. In addition, some oil components do not react with the derivatizing reagent and therefore do not emit a visible color. While some may be seen under UV light, others may not be visible at all. Another complicating factor is that compounds found in trace amounts may fall under the detection limit of the HPTLC instrument. A more complex mixture of coeluting chemicals also represents a challenge. Two or more developments may be required for better separation of these target constituents.

New studies are currently in process, involving two-dimensional and multigradient developments to address the above-mentioned challenges. Future work prospects include the addition of a densitometry module and a TLC-MS interface to achieve a more accurate quantification and precise recovery of individual oil components for further analysis. With so many favorable features and few obstacles, this technique proved to be an efficient and reliable screening tool for the selected *Melaleuca* oils.

## Figures and Tables

**Table 1:** Species, sample codes, and sources of oils used in this study.

Species	Code	Source
*M. alternifolia* (Maiden & Betche) Cheel	TT_AA_	Aromappeal (Puritan’s Pride, Inc.), Oakdale, NY, USA
*M. alternifolia* (Maiden & Betche) Cheel	TT_AS_	Apothecary Shoppe, Portland, OR, USA
*M. alternifolia* (Maiden & Betche) Cheel	TT_EG_	Eden’s Garden, San Clemente, CA, USA
*M. alternifolia* (Maiden & Betche) Cheel	TT_FC_	Floracopeia, Grass Valley, CA, USA
*M. alternifolia* (Maiden & Betche) Cheel	TT_NG_	Nature’s Gift, Madison, TN, USA
*M. alternifolia* (Maiden & Betche) Cheel	TT_PT_	Plant Therapy, Inc, Twin Falls, ID, USA
*M. alternifolia* (Maiden & Betche) Cheel	TT_SAT_	SAT Group, Kannauj, India
*M. cajuputi* Powell	M_Ca_	Nature’s Gift, Madison, TN, USA
*M. quinquenervia* (Cav.) S.T. Blake	M_Ne_	Nature’s Gift, Madison, TN, USA
*M. quinquenervia* (Cav.) S.T. Blake	M_Ni_	Nature’s Gift, Madison, TN, USA
*M. ericifolia* Sm.	M_Ro_	Nature’s Gift, Madison, TN, USA

**Table 2:** Comparative percentage composition of the *Melaleuca* EOs.

#	* RI Exp	** RILit	Compounds	TT_AA_	TT_AS_	TT_EG_	TT_FC_	TT_NG_	TT_PT_	TT_SAT_	M_Ca_	M_Ne_	M_Ni_	M_Ro_
**1**	938	930	α-Thujene ^RI, MS^	0.85 ± 0.10	0.77 ± 0.05	0.76 ± 0.04	0.48 ± 0.02	0.97 ± 0.06	0.79 ± 0.01	0.30 ± 0.10	0.77 ± 0.10	0.27 ± 0.03	1.07 ± 0.28	0.17 ± 0.00
**2**	946	939	α-Pinene ^RI, MS, Std^	1.89 ± 0.06	2.66 ± 0.03	2.40 ± 0.41	1.67 ± 0.05	2.48 ± 0.16	1.39 ± 0.02	1.29 ± 0.16	5.48 ± 0.11	0.57 ± 0.12	9.75 ± 0.13	5.09 ± 0.03
**3**	954	954	Camphene ^RI, MS^	0.00	0.00	0.00	0.00	0.00	0.01 ± 0.00	0.01 ± 0.00	0.15 ± 0.02	0.00	0.04 ± 0.02	0.28 ± 0.05
**4**	969	960	Benzaldehyde ^RI, MS, Std^	0.00	0.00	0.00	0.00	0.00	0.00	0.00	0.00	0.38 ± 0.04	0.09 ± 0.04	0.00
**5**	970	975	Sabinene ^RI, MS, Std^	0.72 ± 0.03	0.05 ± 0.00	0.36 ± 0.04	0.45 ± 0.06	0.94 ± 0.03	0.64 ± 0.07	0.08 ± 0.02	0.18 ± 0.04	0.00	0.00	0.00
**6**	989	979	β-pinene ^RI, MS, Std^	0.73 ± 0.01	0.88 ± 0.07	0.89 ± 0.10	0.97 ± 0.16	1.06 ± 0.02	0.80 ± 0.08	0.93 ± 0.01	1.11 ± 0.07	0.94 ± 0.08	2.56 ± 0.33	0.64 ± 0.03
**7**	998	990	Myrcene ^RI, MS, Std^	0.48 ± 0.06	0.58 ± 0.05	0.63 ± 0.04	0.62 ± 0.12	0.77 ± 0.03	0.41 ± 0.06	0.63 ± 0.02	0.65 ± 0.03	0.60 ± 0.16	0.72 ± 0.20	0.61 ± 0.02
**8**	1009	991	6-Methyl-5-hepten-2-ol ^RI, MS, Std^	0.00	0.00	0.00	0.00	0.00	0.00	0.00	0.00	0.19 ± 0.03	0.00	0.00
**9**	1010	1002	α-Phellandrene ^RI, MS, Std^	0.14 ± 0.07	0.54 ± 0.02	0.45 ± 0.07	0.47 ± 0.10	0.53 ± 0.08	0.33 ± 0.10	0.43 ± 0.13	0.50 ± 0.02	0.09 ± 0.01	0.11 ± 0.03	0.03 ± 0.01
**10**	1012	1003	Pseudolimonene ^RI, MS^	0.01 ± 0.00	0.01 ± 0.00	0.01 ± 0.00	0.01 ± 0.00	0.01 ± 0.00	0.01 ± 0.00	0.00	0.00	0.00	0.00	0.00
**11**	1018	1011	δ-3-Carene ^RI, MS, Std^	0.00	0.00	0.00	0.00	0.00	0.00	0.00	0.00	0.00	0.00	0.20 ± 0.04
**12**	1021	1014	1,4-Cineole ^RI, MS, Std^	0.00	0.00	0.04 ± 0.01	0.00	0.00	0.00	0.00	0.00	0.00	0.00	0.00
**13**	1022	1017	α-Terpinene ^RI, MS, Std^	6.14 ± 0.03	12.62 ± 0.41	9.62 ± 0.14	8.35 ± 0.13	8.89 ± 0.37	7.63 ± 0.19	3.47 ± 0.06	0.22 ± 0.01	0.13 ± 0.03	0.00	0.17 ± 0.04
**14**	1030	1024	*p*-Cymene ^RI, MS, Std^	5.18 ± 0.55	1.66 ± 0.15	3.51 ± 0.04	3.59 ± 0.27	3.22 ± 0.28	3.71 ± 0.17	11.47 ± 0.19	0.79 ± 0.11	0.03 ± 0.02	0.07 ± 0.02	1.21 ± 0.06
**15**	1039	1029	Limonene ^RI, MS, Std^	1.63 ± 0.23	0.30 ± 0.09	1.10 ± 0.07	1.15 ± 0.02	0.87 ± 0.03	1.10 ± 0.05	0.51 ± 0.19	0.10 ± 0.00	0.19 ± 0.03	2.83 ± 0.18	3.03 ± 0.05
**16**	1039	1029	β-Phellandrene ^RI, MS, Std^	0.10 ± 0.01	0.26 ± 0.01	0.86 ± 0.04	0.83 ± 0.06	0.03 ± 0.00	0.44 ± 0.04	0.39 ± 0.01	0.07 ± 0.01	0.01 ± 0.00	0.01 ± 0.00	0.00
**17**	1040	1031	1,8-Cineole ^RI, MS, Std^	2.85 ± 0.38	1.17 ± 0.01	3.76 ± 0.12	3.28 ± 0.07	4.79 ± 0.14	3.90 ± 0.08	4.50 ± 0.09	64.63 ± 1.19	1.91 ± 0.16	52.20 ± 0.28	27.57 ± 0.56
**18**	1049	1037	(*Z*)-β-Ocimene ^RI, MS, Std^	0.00	0.00	0.00	0.00	0.00	0.00	0.00	0.00	0.69 ± 0.10	0.00	0.04 ± 0.01
**19**	1062	1050	(*E*)-β-Ocimene ^RI, MS, Std^	0.01 ± 0.00	0.01 ± 0.00	0.01 ± 0.00	0.01 ± 0.00	0.01 ± 0.00	0.01 ± 0.00	0.00	0.00	0.74 ± 0.10	0.03 ± 0.01	0.76 ± 0.08
**20**	1071	1059	γ-Terpinene ^RI, MS, Std^	16.42 ± 0.83	18.91 ± 0.46	19.58 ± 0.10	19.60 ± 0.10	16.58 ± 0.45	20.75 ± 0.20	16.61 ± 0.41	3.24 ± 0.20	0.12 ± 0.05	0.57 ± 0.00	1.84 ± 0.01
**21**	1081	1070	*cis*-Sabinene hydrate ^RI, MS^	0.00	0.01 ± 0.00	0.14 ± 0.04	0.20 ± 0.09	0.29 ± 0.07	0.07 ± 0.02	0.09 ± 0.01	0.01 ± 0.01	0.00	0.00	0.00
**22**	1084	1072	*cis*-Linalool oxide ^RI, MS, Std^	0.00	0.00	0.30 ± 0.14	0.00	0.00	0.00	0.00	0.00	0.07 ± 0.00	0.00	0.13 ± 0.13
**23**	1098	1088	Terpinolene ^RI, MS, Std^	2.83 ± 0.05	3.61 ± 0.01	4.19 ± 0.04	3.63 ± 0.21	3.39 ± 0.12	3.16 ± 0.06	2.75 ± 0.04	1.04 ± 0.05	0.26 ± 0.10	0.45 ± 0.07	2.14 ± 0.02
**24**	1107	1096	Linalool ^RI, MS, Std^	0.07 ± 0.02	0.13 ± 0.01	0.45 ± 0.15	1.12 ± 0.03	1.04 ± 0.04	0.13 ± 0.06	0.22 ± 0.02	0.26 ± 0.04	33.30± 0.63	0.47 ± 0.29	38.19 ± 0.06
**25**	1115	1097	Hotrienol ^RI, MS^	0.00	0.00	0.01 ± 0.00	0.01 ± 0.00	0.01 ± 0.00	0.01 ± 0.00	0.00	0.00	0.12 ± 0.06	0.00	0.15 ± 0.02
**26**	1123	1121	*cis*-*p*-Menth-2-en-1-ol	0.14 ± 0.08	0.21 ± 0.06	0.21 ± 0.05	0.00	0.37 ± 0.08	0.28 ± 0.01	0.36 ± 0.05	0.00	0.00	0.00	0.00
**27**	1125	1122	*trans*-*p*-Menth-2-en-1-ol	0.21 ± 0.01	0.18 ± 0.01	0.17 ± 0.02	0.15 ± 0.02	0.31 ± 0.04	0.22 ± 0.01	0.28 ± 0.04	0.00	0.00	0.00	0.00
**28**	1168	1166	δ-Terpinol ^RI, MS^	0.00	0.00	0.00	0.00	0.00	0.00	0.00	0.12 ± 0.01	0.00	0.11 ± 0.01	0.12 ± 0.01
**29**	1183	1177	Terpinene-4-ol ^RI, MS, Std^	44.28 ± 0.90	38.62 ± 0.33	37.66 ± 0.24	40.36 ± 0.53	38.42 ± 0.22	40.55 ± 0.67	39.57 ± 1.37	0.71 ± 0.03	0.27 ± 0.00	0.62 ± 0.01	0.79 ± 0.01
**30**	1188	1182	*p*-Cymen-8-ol ^RI, MS^	0.14 ± 0.04	0.05 ± 0.02	0.12 ± 0.00	0.17 ± 0.03	0.11 ± 0.01	0.10 ± 0.02	0.19 ± 0.10	0.02 ± 0.01	0.00	0.00	0.10 ± 0.01
**31**	1198	1188	α-Terpineol ^RI, MS, Std^	3.74 ± 0.05	3.11 ± 0.01	3.35 ± 0.07	3.61 ± 0.09	4.66 ± 0.03	3.40 ± 0.04	4.20 ± 0.09	5.44 ± 0.10	0.46 ± 0.01	5.08 ± 0.06	4.05 ± 0.08
**32**	1206	1196	*cis*-Piperitol ^RI, MS, Std^	0.08 ± 0.02	0.07 ± 0.00	0.07 ± 0.00	0.03 ± 0.00	0.08 ± 0.01	0.09 ± 0.01	0.13 ± 0.02	0.00	0.00	0.00	0.00
**33**	1218	1208	*trans*-Piperitol ^RI, MS^	0.14 ± 0.01	0.09 ± 0.01	0.10 ± 0.00	0.05 ± 0.00	0.08 ± 0.00	0.15 ± 0.00	0.14 ± 0.06	0.00	0.00	0.00	0.00
**34**	1226	1216	*trans*-Carveol ^RI, MS,^	0.00	0.00	0.00	0.00	0.00	0.00	0.00	0.00	0.00	0.00	0.01 ± 0.01
**35**	1232	1221	*cis*-Sabinene hydrate acetate ^RI, MS^	0.00	0.00	0.00	0.00	0.00	0.00	0.00	0.00	0.08 ± 0.00	0.00	0.00
**36**	1236	1225	Citronellol ^RI, MS, Std^	0.00	0.00	0.00	0.00	0.00	0.00	0.00	0.00	0.00	0.01 ± 0.00	0.02 ± 0.00
**37**	1238	1229	Nerol ^RI, MS^	0.00	0.00	0.00	0.00	0.00	0.00	0.00	0.00	0.01 ± 0.00	0.00	0.16 ± 0.01
**38**	1243	1237	Ascaridole ^RI, MS^	0.00	0.00	0.00	0.00	0.00	0.00	0.05 ± 0.03	0.00	0.00	0.00	0.00
**39**	1245	1238	Neral	0.00	0.00	0.00	0.00	0.00	0.00	0.00	0.00	0.00	0.00	0.09 ± 0.01
**40**	1253	1252	Geraniol ^RI, MS, Std^	0.05 ± 0.05	0.00	0.04 ± 0.03	0.02 ± 0.00	0.03 ± 0.00	0.00	0.02 ± 0.02	0.04 ± 0.02	0.19 ± 0.02	0.02 ± 0.01	0.20 ± 0.00
**41**	1264	1258	2-Phenyl ethyl acetate ^RI, MS^	0.00	0.00	0.00	0.00	0.00	0.00	0.00	0.00	0.00	0.00	0.08 ± 0.02
**42**	1271	1267	Geranial ^RI, MS^	0.00	0.00	0.00	0.00	0.00	0.00	0.00	0.00	0.00	0.00	0.12 ± 0.01
**43**	1273	1269	*trans*-Ascaridol glycol ^RI, MS^	0.12 ± 0.02	0.01 ± 0.00	0.04 ± 0.00	0.05 ± 0.02	0.02 ± 0.00	0.04 ± 0.01	0.06 ± 0.02	0.00	0.00	0.00	0.00
**44**	1291	1288	*cis*-Ascaridol glycol ^RI, MS^	0.04 ± 0.02	0.01 ± 0.00	0.02 ± 0.00	0.04 ± 0.01	0.00	0.01 ± 0.00	0.04 ± 0.01	0.00	0.00	0.00	0.00
**45**	1295	1290	Thymol ^RI, MS, Std^	0.00	0.00	0.00	0.01 ± 0.00l	0.00	0.00	0.02 ± 0.01	0.00	0.00	0.00	0.00
**46**	1306	1299	Carvacrol ^RI, MS, Std^	0.01 ± 0.01	0.00	0.02 ± 0.01	0.02 ± 0.01	0.00	0.00	0.02 ± 0.01	0.00	0.00	0.00	0.00
**47**	1330	1324	Methyl geranate ^RI, MS^	0.00	0.00	0.00	0.00	0.00	0.00	0.00	0.00	0.00	0.00	0.07 ± 0.03
**48**	1343	1338	δ-Elemene ^RI, MS^	0.01 ± 0.00	0.01 ± 0.00	0.01 ± 0.00	0.00	0.05 ± 0.00	0.01 ± 0.00	0.02 ± 0.01	0.01 ± 0.00	0.00	0.00	0.02 ± 0.00
**49**	1355	1347	α-Terpinyl acetate ^RI, MS, Std^	0.00	0.00	0.00	0.00	0.00	0.00	0.00	0.64 ± 0.01	0.00	1.30 ± 0.02	0.00
**50**	1356	1348	α-Cubebene ^RI, MS^	0.01 ± 0.01	0.04 ± 0.00	0.03 ± 0.00	0.01 ± 0.00	0.07 ± 0.00	0.03 ± 0.00	0.06 ± 0.02	0.00	0.00	0.00	0.00
**51**	1359	1359	Eugenol ^RI, MS, Std^	0.00	0.00	0.00	0.00	0.00	0.00	0.00	0.00	0.01 ± 0.00	0.00	0.00
**52**	1376	1375	α-Ylangene ^RI, MS^	0.00	0.00	0.00	0.00	0.00	0.00	0.00	0.13 ± 0.02	0.00	0.00	0.00
**53**	1378	1376	Isoledene ^RI, MS^	0.06 ± 0.01	0.08 ± 0.01	0.03 ± 0.01	0.04 ± 0.00	0.06 ± 0.00	0.05 ± 0.01	0.06 ± 0.02	0.00	0.00	0.00	0.15 ± 0.02
**54**	1380	1376	α-Copaene ^RI, MS, Std^	0.10 ± 0.01	0.14 ± 0.01	0.10 ± 0.01	0.07 ± 0.00	0.13 ± 0.00	0.06 ± 0.02	0.13 ± 0.02	0.08 ± 0.01	0.00	0.03 ± 0.00	0.02 ± 0.01
**55**	1382	1381	Geranyl acetate ^RI, MS, Std^	0	0	0	0	0	0	0	0	0.00	0.00	0.02 ± 0.01
**56**	1395	1390	β-Elemene ^RI, MS, Std^	0.03 ± 0.01	0.01 ± 0.00	0.02 ± 0.00	0.01 ± 0.00	0.06 ± 0.00	0.01 ± 0.00	0.03 ± 0.01	0.05 ± 0.03	0.00	0.00	0.32 ± 0.02
**57**	1403	1402	α-Funebrene ^RI, MS, Std^	0.00	0.00	0.00	0.00	0.00	0.00	0.00	0.00	0.03 ± 0.01	0.00	0.00
**58**	1404	1403	Methyl eugenol ^RI, MS, Std^	0.00	0.00	0.01 ± 0.00	0.01 ± 0.01	0.00	0.00	0.02 ± 0.00	0.00	0.00	0.00	0.00
**59**	1409	1408	Isocaryophyllene ^RI, MS^	0.00	0.00	0.00	0.00	0.00	0.00	0.00	0.01 ± 0.01	0.02 ± 0.01	0.00	0.00
**60**	1410	1409	α-Gurjunene ^RI, MS^	0.41 ± 0.01	0.58 ± 0.01	0.36 ± 0.01	0.18 ± 0.06	0.45 ± 0.01	0.35 ± 0.01	0.43 ± 0.06	0.06 ± 0.03	0.00	0.07 ± 0.02	0.39 ± 0.01
**61**	1412	1411	α-Cedrene ^RI, MS^	0.00	0.00	0.00	0.00	0.00	0.00	0.00	0.00	0.06 ± 0.03	0.00	0.00
**62**	1417	1416	β-Maaliene ^RI, MS^	0.01 ± 0.00	0.02 ± 0.00	0.02 ± 0.00	0.00	0.01 ± 0.00	0.00	0.02 ± 0.00	0.00	0.00	0.00	0.03 ± 0.01
**63**	1421	1419	β-Caryophyllene ^RI, MS, Std^	0.37 ± 0.01	0.74 ± 0.02	0.29 ± 0.01	0.21 ± 0.01	0.56 ± 0.02	0.28 ± 0.03	0.40 ± 0.04	3.82 ± 0.12	2.40 ± 0.05	2.66 ± 0.05	0.16 ± 0.01
**64**	1429	1425	γ-Maaliene ^RI, MS^	0.06 ± 0.01	0.08 ± 0.00	0.07 ± 0.00	0.05 ± 0.01	0.06 ± 0.00	0.04 ± 0.00	0.06 ± 0.01	0.01 ± 0.01	0.00	0.01 ± 0.01	0.10 ± 0.01
**65**	1433	1433	β-Gurjunene ^RI, MS^	0.01 ± 0.00	0.01 ± 0.00	0.01 ± 0.00	0.00	0.01 ± 0.00	0.00	0.02 ± 0.00	0.05 ± 0.01	0.00	0.00	0.10 ± 0.00
**66**	1435	1433	α-Maaliene ^RI, MS, Std^	0.07 ± 0.01	0.09 ± 0.00	0.07 ± 0.01	0.05 ± 0.01	0.07 ± 0.00	0.05 ± 0.00	0.07 ± 0.02	0.05 ± 0.01	0.00	0.00	0.12 ± 0.01
**67**	1439	1441	Aromadendrene ^RI, MS, Std^	1.51 ± 0.02	1.90 ± 0.02	1.20 ± 0.03	1.25 ± 0.04	1.24 ± 0.01	1.51 ± 0.19	1.27 ± 0.09	0.65 ± 0.04	0.00	0.16 ± 0.08	3.38 ± 0.02
**68**	1443	1443	Selina-5,11-diene ^RI, MS^	0.16 ± 0.01	0.22 ± 0.01	0.14 ± 0.01	0.08 ± 0.04	0.17 ± 0.00	0.11 ± 0.01	0.15 ± 0.02	0.06 ± 0.01	0.00	0.00	0.26 ± 0.01
**69**	1448	1451	Amorpha-4,11-diene ^RI, MS^	0.00	0.00	0.00	0.00	0.00	0.00	0.00	0.00	0.07 ± 0.03	0.00	0.00
**70**	1450	1453	*trans*-Muurola-3.5-diene ^RI, MS^	0.11 ± 0.01	0.19 ± 0.01	0.12 ± 0.01	0.03 ± 0.00	0.31 ± 0.01	0.14 ± 0.01	0.18 ± 0.03	0.02 ± 0.01	0.00	0.01 ± 0.00	0.06 ± 0.01
**71**	1453	1454	α-Humulene ^RI, MS, Std^	0.08 ± 0.00	0.12 ± 0.00	0.07 ± 0.00	0.05 ± 0.00	0.15 ± 0.02	0.07 ± 0.01	0.09 ± 0.01	1.81 ± 0.08	0.40 ± 0.01	0.40 ± 0.03	0.03 ± 0.00
**72**	1457	1456	(*E*)-β-Farnesene ^RI, MS, Std^	0.00	0.00	0.00	0.00	0.00	0.00	0.00	0.00	1.93 ± 0.40	0.12 ± 0.05	0.00
**73**	1459	1460	Alloaromadendrene ^RI, MS, Std^	0.67 ± 0.01	0.96 ± 0.01	0.53 ± 0.02	0.47 ± 0.02	0.62 ± 0.01	0.54 ± 0.01	0.62 ± 0.05	0.36 ± 0.02	0.00	0.51 ± 0.05	1.41 ± 0.02
**74**	1464	1466	α-Acoradiene ^RI, MS^	0.00	0.00	0.00	0.00	0.00	0.00	0.00	0.00	0.01 ± 0.00	0.00	0.00
**75**	1467	1470	β-Acoradiene ^RI, MS^	0.00	0.00	0.00	0.00	0.00	0.00	0.00	0.00	0.03 ± 0.01	0.00	0.00
**76**	1471	1475	10-*epi*-β-Acoradiene ^RI, MS^	0.00	0.00	0.00	0.00	0.00	0.00	0.00	0.00	0.02 ± 0.01	0.00	0.00
**77**	1473	1476	*trans*-Cadina-1(6),4-diene ^RI, MS^	0.36 ± 0.01	0.55 ± 0.02	0.36 ± 0.01	0.20 ± 0.01	0.45 ± 0.02	0.42 ± 0.02	0.45 ± 0.05	0.00	0.02 ± 0.01	0.00	0.00
**78**	1474	1477	γ-Gurjunene ^RI, MS^	0.00	0.00	0.00	0.00	0.00	0.00	0.00	0.00	0.00	0.00	0.35 ± 0.01
**79**	1476	1479	γ-Muurolene ^RI, MS^	0.04 ± 0.00	0.05 ± 0.00	0.04 ± 0.00	0.03 ± 0.01	0.04 ± 0.00	0.03 ± 0.00	0.05 ± 0.01	0.45 ± 0.03	0.00	0.07 ± 0.01	0.08 ± 0.01
**80**	1480	1480	*ar*-Curcumene ^RI, MS, Std^	0.00	0.00	0.00	0.00	0.00	0.00	0.00	0.00	0.13 ± 0.04	0.00	0.00
**81**	1481	1482	γ-Curcumene ^RI, MS^	0.00	0.00	0.00	0.00	0.00	0.00	0.00	0.00	0.09 ± 0.02	0.00	0.00
**82**	1483	1484	α-Amorphene ^RI, MS^	0.00	0.00	0.00	0.00	0.00	0.00	0.00	0.30 ± 0.01	0.00	0.00	0.00
**83**	1484	1485	Germacrene D ^RI, MS^	0.01 ± 0.00	0.01 ± 0.00	0.01 ± 0.00	0.01 ± 0.00	0.01 ± 0.00	0.01 ± 0.00	0.01 ± 0.00	0.00	0.00	0.18 ± 0.02	0.00
**84**	1486	1490	β-Selinene ^RI, MS^	0.09 ± 0.01	0.11 ± 0.01	0.10 ± 0.01	0.09 ± 0.01	0.06 ± 0.00	0.05 ± 0.01	0.08 ± 0.02	1.28 ± 0.05	0.00	0.20 ± 0.01	0.23 ± 0.01
**85**	1488	1490	Alloaromadendr-9-ene ^RI, MS^	0.11 ± 0.01	0.14 ± 0.01	0.07 ± 0.04	0.08 ± 0.01	0.11 ± 0.00	0.07 ± 0.01	0.11 ± 0.02	0.00	0.00	0.18 ± 0.00	0.29 ± 0.01
**86**	1491	1492	δ-Selinene ^RI, MS^	0.00	0.00	0.00	0.00	0.00	0.00	0.00	0.35 ± 0.01	0.00	0.00	0.00
**87**	1492	1493	*cis*-β-Guaiene ^RI, MS^	0.19 ± 0.01	0.28 ± 0.01	0.18 ± 0.01	0.11 ± 0.01	0.31 ± 0.01	0.16 ± 0.01	0.21 ± 0.03	0.11 ± 0.01	0.00	0.01 ± 0.01	0.16 ± 0.01
**88**	1494	1496	Ledene ^RI, MS^	1.14 ± 0.01	1.62 ± 0.02	0.86 ± 0.03	0.47 ± 0.03	1.06 ± 0.01	1.38 ± 0.05	1.69 ± 0.05	0.19 ± 0.03	0.16 ± 0.06	1.76 ± 0.17	0.98 ± 0.02
**89**	1495	1498	α-Selinene ^RI, MS^	0.00	0.00	0.00	0.00	0.00	0.00	0.00	1.12 ± 0.08	0.00	0.00	0.00
**90**	1496	1499	(*Z*,*E*)-α-Farnesene ^RI, MS^	0.00	0.00	0.00	0.00	0.00	0.00	0.00	0.00	0.64 ± 0.14	0.00	0.00
**91**	1497	1500	Bicyclogermacrene ^RI, MS^	0.91 ± 0.01	0.96 ± 0.06	0.67 ± 0.02	0.31 ± 0.01	0.72 ± 0.02	0.91 ± 0.04	0.60 ± 0.03	0.00	0.00	0.02 ± 0.01	0.62 ± 0.02
**92**	1498	1500	α-Muurolene ^RI, MS^	0.17 ± 0.01	0.20 ± 0.00	0.14 ± 0.00	0.11 ± 0.01	0.22 ± 0.00	0.16 ± 0.01	0.20 ± 0.02	0.06 ± 0.01	0.00	0.09 ± 0.01	0.03 ± 0.00
**93**	1501	1501	Epizonarene ^RI, MS^	0.02 ± 0.00	0.03 ± 0.00	0.02 ± 0.00	0.01 ± 0.00	0.03 ± 0.00	0.01 ± 0.00	0.02 ± 0.00	0.19 ± 0.01	0.00	0.00	0.00
**94**	1503	1502	*trans*-β-Guaiene ^RI, MS^	0.00	0.00	0.00	0.00	0.00	0.00	0.00	0.00	0.09 ± 0.07	0.00	0.00
**95**	1505	1505	(*Z*)-α-Bisabolene ^RI, MS^	0.00	0.00	0.00	0.00	0.00	0.00	0.00	0.00	0.23 ± 0.03	0.00	0.00
**96**	1506	1505	(*E*,*E*)-α-Farnesene ^RI, MS, Std^	0.00	0.00	0.00	0.00	0.00	0.00	0.00	0.00	1.67 ± 0.38	0.11 ± 0.04	0.00
**97**	1508	1512	δ-Amorphene ^RI, MS^	0.00	0.00	0.00	0.00	0.00	0.00	0.00	0.00	0.00	0.00	0.19 ± 0.01
**98**	1512	1513	γ-Cadinene ^RI, MS^	0.01 ± 0.00	0.02 ± 0.00	0.02 ± 0.00	0.01 ± 0.00	0.02 ± 0.00	0.01 ± 0.00	0.03 ± 0.00	0.07 ± 0.01	0.00	0.25 ± 0.02	0.06 ± 0.01
**99**	1514	1515	(*Z*)-γ-Bisabolene ^RI, MS^	0.00	0.00	0.00	0.00	0.00	0.00	0.00	0.00	0.21 ± 0.05	0.00	0.00
**100**	1517	1522	7-*epi*-α-Selinene ^RI, MS^	0.00	0.00	0.00	0.00	0.00	0.00	0.00	0.02 ± 0.01	0.00	0.00	0.06 ± 0.01
**101**	1518	1522	*trans*-Calamene ^RI, MS^	0.07 ± 0.06	0.01 ± 0.00	0.01 ± 0.00	0.01 ± 0.00	0.00	0.01 ± 0.00	0.00	0.00	0.00	0.00	0.00
**102**	1521	1523	δ-Cadinene ^RI, MS^	1.35 ± 0.01	1.71 ± 0.02	1.22 ± 0.04	1.06 ± 0.04	1.32 ± 0.02	1.78 ± 0.14	1.79 ± 0.09	0.23 ± 0.03	0.13 ± 0.04	0.35 ± 0.01	0.17 ± 0.03
**103**	1527	1529	Zonarene ^RI, MS^	0.75 ± 0.02	0.58 ± 0.04	0.33 ± 0.02	0.30 ± 0.01	0.37 ± 0.02	0.54 ± 0.04	0.48 ± 0.06	0.04 ± 0.01	0.04 ± 0.01	0.00	0.03 ± 0.02
**104**	1529	1531	(*E*)-γ-Bisabolene ^RI, MS^	0.00	0.00	0.00	0.00	0.00	0.00	0.00	0.00	0.19 ± 0.05	0.00	0.00
**105**	1531	1534	*trans*-Cadina-1,4-diene	0.22 ± 0.01	0.28 ± 0.01	0.19 ± 0.01	0.14 ± 0.01	0.29 ± 0.01	0.24 ± 0.01	0.27 ± 0.02	0.00	0.00	0.00	0.00
**106**	1535	1538	α-Cadinene ^RI, MS^	0.00	0.00	0.00	0.00	0.00	0.00	0.00	0.06 ± 0.03	0.00	0.00	0.01 ± 0.00
**107**	1540	1545	α-Calacorene ^RI, MS^	0.01 ± 0.00	0.01 ± 0.00	0.02 ± 0.00	0.02 ± 0.01	0.00	0.01 ± 0.00	0.04 ± 0.03	0.00	0.00	0.00	0.03 ± 0.03
**108**	1542	1546	Selina-3,7(11)-diene ^RI, MS^	0.00	0.00	0.01 ± 0.00	0.00	0.00	0.01 ± 0.00	0.00	0.16 ± 0.02	0.00	0.00	0.00
**109**	1548	1547	(*E*)-α-Bisabolene ^RI, MS^	0.00	0.00	0.00	0.00	0.00	0.00	0.00	0.00	0.41 ± 0.11	0.00	0.00
**110**	1559	1561	Germacrene B ^RI, MS^	0.00	0.00	0.00	0.00	0.00	0.00	0.00	0.11 ± 0.01	0.00	0.00	0.00
**111**	1560	1563	(*E*)-Nerolidol ^RI, MS, Std^	0.00	0.00	0.00	0.00	0.00	0.00	0.00	0.00	48.40 ± 1.21	6.89 ± 0.44	0.00
**112**	1562	1567	Maaliol ^RI, MS^	0.06 ± 0.01	0.06 ± 0.01	0.06 ± 0.01	0.13 ± 0.01	0.03 ± 0.00	0.01 ± 0.01	0.01 ± 0.00	0.00	0.00	0.00	0.00
**113**	1563	1568	Palustrol ^RI, MS^	0.07 ± 0.01	0.07 ± 0.01	0.09 ± 0.01	0.19 ± 0.02	0.04 ± 0.00	0.02 ± 0.01	0.08 ± 0.02	0.01 ± 0.00	0.00	0.00	0.09 ± 0.01
**114**	1572	1578	Spathulenol ^RI, MS^	0.12 ± 0.02	0.07 ± 0.01	0.07 ± 0.01	0.14 ± 0.01	0.05 ± 0.00	0.03 ± 0.01	0.10 ± 0.05	0.01 ± 0.00	0.00	0.00	0.25 ± 0.01
**115**	1578	1583	Caryophyllene oxide ^RI, MS, Std^	0.00	0.00	0.00	0.00	0.00	0.00	0.00	0.01 ± 0.00	0.20 ± 0.05	0.00	0.00
**116**	1585	1590	Globulol ^RI, MS, Std^	0.40 ± 0.02	0.43 ± 0.02	0.35 ± 0.02	0.94 ± 0.04	0.25 ± 0.01	0.22 ± 0.01	0.23 ± 0.02	0.38 ± 0.03	0.31 ± 0.04	0.35 ± 0.01	0.59 ± 0.02
**117**	1586	1592	Viridiflorol ^RI, MS, Std^	0.19 ± 0.01	0.17 ± 0.01	0.18 ± 0.01	0.41 ± 0.02	0.10 ± 0.00	0.11 ± 0.01	0.16 ± 0.03	0.40 ± 0.02	0.07 ± 0.01	6.23 ± 0.17	0.15 ± 0.01
**118**	1588	1595	Cubeban-11-ol ^RI, MS^	0.15 ± 0.01	0.16 ± 0.01	0.13 ± 0.01	0.35 ± 0.01	0.07 ± 0.01	0.08 ± 0.01	0.12 ± 0.02	0.00	0.00	0.00	0.12 ± 0.01
**119**	1594	1600	Rosiflorol ^RI, MS^	0.11 ± 0.01	0.12 ± 0.01	0.12 ± 0.01	0.29 ± 0.01	0.06 ± 0.00	0.04 ± 0.01	0.10 ± 0.02	0.00	0.00	0.00	0.10 ± 0.01
**120**	1597	1600	Guaiol ^RI, MS^	0.00	0.00	0.00	0.00	0.00	0.00	0.00	0.03 ± 0.01	0.01 ± 0.00	0.09 ± 0.01	0.11 ± 0.01
**121**	1599	1602	Ledol ^RI, MS^	0.01 ± 0.00	0.01 ± 0.00	0.01 ± 0.00	0.01 ± 0.00	0.01 ± 0.00	0.03 ± 0.02	0.01 ± 0.00	0.05 ± 0.01	0.01 ± 0.00	0.72 ± 0.02	0.03 ± 0.00
**122**	1609	1607	5-*epi*-7-*epi*-α-Eudesmol ^RI, MS^	0.11 ± 0.01	0.13 ± 0.01	0.12 ± 0.01	0.30 ± 0.02	0.04 ± 0.00	0.04 ± 0.01	0.08 ± 0.02	0.00	0.00	0.00	0.14 ± 0.01
**123**	1611	1608	Humulene epoxide II ^RI, MS^	0.00	0.00	0.00	0.00	0.00	0.00	0.00	0.02 ± 0.01	0.02 ± 0.00	0.02 ± 0.01	0.00
**124**	1619	1619	1,10-di-*epi*-Cubenol ^RI, MS^	0.20 ± 0.01	0.22 ± 0.01	0.19 ± 0.01	0.40 ± 0.02	0.14 ± 0.00	0.14 ± 0.01	0.22 ± 0.04	0.00	0.00	0.02 ± 0.01	0.00
**125**	1626	1623	10-*epi*-γ-Eudesmol ^RI, MS^	0.00	0.00	0.00	0.00	0.00	0.00	0.00	0.02 ± 0.01	0.00	0.00	0.00
**126**	1630	1628	1-*epi*-Cubenol ^RI, MS^	0.10 ± 0.01	0.10 ± 0.01	0.10 ± 0.01	0.17 ± 0.01	0.07 ± 0.00	0.06 ± 0.01	0.15 ± 0.01	0.00	0.00	0.06 ± 0.03	0.01 ± 0.00
**127**	1632	1631	Muurola-4,10(14)-dien-1β-ol ^RI, MS^	0.00	0.00	0.00	0.00	0.00	0.00	0.00	0.03 ± 0.00	0.02 ± 0.00	0.00	0.00
**128**	1633	1632	γ-Eudesmol ^RI, MS^	0.00	0.00	0.00	0.00	0.00	0.00	0.00	0.15 ± 0.01	0.00	0.00	0.00
**129**	1639	1640	T-Cadinol ^RI, MS^	0.00	0.00	0.00	0.00	0.00	0.00	0.00	0.01 ± 0.00	0.00	0.00	0.00
**130**	1643	1646	α-Muurolol ^RI, MS^	0.06 ± 0.02	0.03 ± 0.01	0.03 ± 0.01	0.07 ± 0.00	0.02 ± 0.00	0.01 ± 0.00	0.03 ± 0.01	0.00	0.00	0.03 ± 0.00	0.02 ± 0.00
**131**	1645	1646	Cubenol ^RI, MS^	0.00	0.01 ± 0.00	0.00	0.02 ± 0.01	0.00	0.00	0.02 ± 0.00	0.00	0.00	0.06 ± 0.01	0.00
**132**	1649	1650	β-Eudesmol ^RI, MS^	0.00	0.00	0.00	0.00	0.00	0.00	0.00	0.10 ± 0.01	0.00	0.00	0.00
**133**	1652	1653	α-Eudesmol ^RI, MS^	0.00	0.00	0.00	0.00	0.00	0.00	0.00	0.18 ± 0.01	0.00	0.01 ± 0.00	0.00
**134**	1668	1671	Bulnesol ^RI, MS^	0.00	0.00	0.00	0.00	0.00	0.00	0.00	0.01 ± 0.00	0.00	0.01 ± 0.00	0.00
**135**	1679	1684	*epi*-α-Bisabolol ^RI, MS^	0.00	0.00	0.00	0.00	0.00	0.00	0.00	0.00	0.02 ± 0.00	0.00	0.00
**136**	1681	1685	α-Bisabolol ^RI, MS, Std^	0.00	0.00	0.00	0.00	0.00	0.00	0.00	0.00	0.03 ± 0.01	0.00	0.00
**137**	1710	1715	(*E*,*Z*)-Farnesol ^RI, MS, Std^	0.00	0.00	0.00	0.00	0.00	0.00	0.00	0.00	0.01 ± 0.01	0.00	0.00
**138**	1720	1723	(*Z*,*E*)-Farnesol ^RI, MS, Std^	0.00	0.00	0.00	0.00	0.00	0.00	0.00	0.00	0.01 ± 0.01	0.00	0.00
			Total	99.70 ± 0.07	99.85 ± 0.04	99.65 ± 0.00	99.84 ± 0.03	99.87 ± 0.01	99.97 ± 0.02	99.73 ± 0.26	99.49 ± 0.37	99.72 ± 0.11	99.79 ± 0.13	99.53 ± 0.05

* RI_exp_: Retention indices (RIs) calculated from the current study; ** RI_lit_: RI from the literature [41,42,43,44,45,46,47,48,49,50,51,52,53,54,55,56,57]. Identification method: ^RI^: retention index; ^MS^: computer matching of the mass spectra libraries and comparison with the literature data; ^Std^: standards compounds were purchased. TTO and samples and their corresponding major components are represented in blue. Other *Melaleuca* oils and their respective major components are highlighted in gray.

**Table 3:** Eigenvalues and percentage of variability and cumulative.

	F1	F2	F3	F4	F5	F6	F7
Eigenvalue	14.92	8.60	4.80	3.85	1.97	1.31	1.17
Variability (%)	39.26	22.62	12.62	10.13	5.20	3.45	3.07
Cumulative (%)	39.26	61.88	74.50	84.63	89.83	93.27	96.35

**Table 4:** Factor loadings, contributions (%), and squared cosine (cos^2^) values. The positive important contributions are highlighted in blue, and the negative important contributions are highlighted in pink. The highest cos^2^ values are highlighted in green.

		Factor Loadings and Contributions (%)		Squared Cosines (cos^2^)	
#	Compounds	F1	F2	F1	F2
**1**	α−Thujene	0.138 (0.129)	−0.649 (4.945)	0.019	**0.425**
**2**	α−Pinene	−0.546 (2.024)	−0.759 (6.695)	0.302	**0.575**
**5**	Sabinene	0.563 (2.191)	−0.055 (0.042)	**0.327**	0.004
**6**	β-Pinene	−0.517 (1.799)	−0.601 (4.208)	0.268	**0.362**
**7**	Myrcene	−0.341 (0.824)	−0.317 (1.311)	**0.123**	0.113
**9**	α-Phellandrene	0.525 (1.832)	−0.237 (0.681)	0.273	0.059
**13**	α-Terpinene	0.853 (4.851)	0.033 (0.010)	**0.724**	0.001
**14**	*p*-Cymene	0.621 (2.579)	0.033 (0.011)	0.385	0.001
**15**	Limonene	−0.157 (0.163)	−0.357 (1.474)	0.024	0.127
**16**	β-Phellandrene	0.539 (1.963)	0.052 (0.036)	0.293	0.003
**17**	1,8-Cineole	−0.685 (3.144)	−0.667 (5.139)	**0.469**	0.442
**18**	(*Z*)-β-Ocimene	−0.561 (2.106)	0.794 (7.323)	0.314	**0.629**
**19**	(*E*)-β-Ocimene	−0.527 (1.866)	0.628 (4.594)	0.278	**0.395**
**20**	γ-Terpinene	0.947 (6.044)	0.013 (0.002)	**0.902**	0.000
**23**	Terpinolene	0.943 (5.940)	−0.001 (0.000)	**0.886**	0.000
**24**	Linalool	−0.502 (1.693)	0.605 (4.263)	0.253	**0.366**
**29**	Terpinene-4-ol	0.948 (6.034)	0.047 (0.024)	**0.900**	0.002
**31**	α-Terpineol	0.074 (0.037)	−0.923 (9.987)	0.006	**0.858**
**49**	α-Terpinyl acetate	−0.637 (2.725)	−0.708 (5.793)	0.407	**0.498**
**60**	α-Gurjunene	0.862 (4.918)	−0.023 (0.008)	**0.734**	0.001
**63**	β-Caryophyllene	−0.833 (4.665)	−0.247 (0.703)	**0.696**	0.060
**67**	Aromadendrene	0.483 (1.556)	0.015 (0.003)	0.232	0.000
**71**	α-Humulene	−0.586 (2.293)	−0.338 (1.318)	**0.342**	0.113
**72**	(*E*)-β-Farnesene	−0.582 (2.268)	0.753 (6.598)	0.338	**0.567**
**73**	Alloaromadendrene	0.373 (0.904)	−0.237 (0.654)	0.135	0.056
**77**	*trans*-Cadina-1(6),4-diene	0.911 (5.546)	0.050 (0.026)	**0.828**	0.002
**84**	β-Selinene	−0.434 (1.261)	−0.469 (2.534)	0.188	0.218
**88**	Ledene	0.431 (1.232)	−0.422 (2.043)	0.184	0.176
**89**	α-Selinene	−0.407 (1.106)	−0.369 (1.568)	0.165	0.135
**90**	(*Z*,*E*)-α-Farnesene	−0.549 (2.022)	0.786 (7.179)	0.302	**0.617**
**91**	Bicyclogermacrene	0.894 (5.351)	0.067 (0.053)	**0.798**	0.005
**96**	(*E*,*E*)-α-Farnesene	−0.584 (2.283)	0.751 (6.563)	0.341	**0.564**
**102**	δ-Cadinene	0.917 (5.655)	−0.024 (0.005)	**0.844**	0.000
**103**	Zonarene	0.873 (5.105)	0.073 (0.065)	**0.762**	0.006
**111**	(*E*)-Nerolidol	−0.620 (2.580)	0.707 (5.811)	0.385	**0.499**
**116**	Globulol	0.016 (0.001)	0.005 (0.000)	0.000	0.000
**117**	Viridiflorol	−0.486 (1.593)	−0.602 (4.188)	0.238	**0.360**
**121**	Ledol	−0.506 (1.718)	−0.599 (4.143)	0.256	**0.356**

**Table 5:** List of UHM * components used in this study.

Label	Name	CAS#
(**e**)	Phthalamide	85-41–6
(**f**)	9-Hydroxyfluorene	1689-64-1
(**g**)	Thioxanthen-9-one	492-22-8
(**h**)	2-(2H-Benzotriazol-2-yl)-4-(1,1,3,3-tetramethylbutyl) phenol	3147-75-9

* UHM components visible under the established analytical conditions.

**Table 6:** System suitability standards (SSTs) and reference standards for *Melaleuca* EOs.

Track	Name	CAS#	Track	Name	CAS#
1	Isoeugenol	97-54-1	11	1,8-Cineole	470-82-6
2	Isoeugenyl acetate	93-29-8	12	(*S*)-(−)-Limonene	5989-54-8
3	(1*R*)-(+)-α-Pinene	7785-70-8	13	(−)-Caryophyllene oxide	1139-30-6
4	α-Phellandrene	99-83-2	14	Nerolidol	7212-44-4
5	Ocimene mix	13877-91-3	15	(−)-Terpinen-4-ol	20126-76-5
6	(+)-Aromadendrene	489-39-4	16	(−)-Linalool	1126-91-0
7	Farnesene mix	502-61-4	17	Viridiflorol	0552-02-03
8	α-Humulene	6753-98-6	18	(−)-α-Terpineol	10482-56-1
9	β-Caryophyllene	87-44-5	19	(−)-Globulol	489-41-8
10	α-Terpinyl acetate	80-26-2	20	Geraniol	106-24-1

## Data Availability

The data are available from the authors upon request.
